# Recent Advances in pH-Responsive Freshness Indicators Using Natural Food Colorants to Monitor Food Freshness

**DOI:** 10.3390/foods11131884

**Published:** 2022-06-25

**Authors:** Danfei Liu, Changfan Zhang, Yumei Pu, Siyuan Chen, Lei Liu, Zijie Cui, Yunfei Zhong

**Affiliations:** School of Packaging and Materials Engineering, Hunan University of Technology, Zhuzhou 412007, China; m18080502003@stu.hut.edu.cn (D.L.); zcf@hut.edu.cn (C.Z.); m21085600005@stu.hut.edu.cn (Y.P.); m20085600011@stu.hut.edu.cn (S.C.); d21080500006@stu.hut.edu.cn (L.L.); m19085204003@stu.hut.edu.cn (Z.C.)

**Keywords:** intelligent packaging technology, natural food colorants, freshness indicator, pH-responsive, food quality

## Abstract

Recently, due to the enhancement in consumer awareness of food safety, considerable attention has been paid to intelligent packaging that displays the quality status of food through color changes. Natural food colorants show useful functionalities (antibacterial and antioxidant activities) and obvious color changes due to their structural changes in different acid and alkali environments, which could be applied to detect these acid and alkali environments, especially in the preparation of intelligent packaging. This review introduces the latest research on the progress of pH-responsive freshness indicators based on natural food colorants and biodegradable polymers for monitoring packaged food quality. Additionally, the current methods of detecting food freshness, the preparation methods for pH-responsive freshness indicators, and their applications for detecting the freshness of perishable food are highlighted. Subsequently, this review addresses the challenges and prospects of pH-responsive freshness indicators in food packaging, to assist in promoting their commercial application.

## 1. Introduction

Packaging is usually used to protect goods from environmental pollution and other effects (such as odors, vibrations, dust, physical damage, temperature, light, and humidity) and to provide consumers with commodity information such as production date, shelf life, nutrients, and usages [1]. It is crucial for guaranteeing food quality and safety and for contributing to prolonging shelf life and reducing food loss and waste [2,3,4]. Recently, with the improvement of living standards and the gradual enhancement of consumer health awareness, food safety issues have attracted considerable attention, further promoting the development of new packaging technologies. Traditional food packaging is mainly used as a protective barrier to resist external forces and to promote sales [5]. Oxidation, microbial growth, and decomposition of enzymes are the main causes of spoilage in many foods (such as grapes, pears, fish, pork, and shrimp) during production, transportation, processing, storage, and sale [6,7]. These processes are directly linked to the loss of food quality and safety, affecting consumers’ health and the overall economy of the food industry [8]. Hence, protecting food quality is an important research orientation since it directly affects the goal of improving quality of life. Additionally, the improvement in awareness of food safety for consumers leads to higher requirements for food safety. Intelligent packaging improves product quality and safety while promoting product sales and enhancing the influence of a company [9,10]. Intelligent packaging involves intelligent devices that can detect the quality of packaged food or internal packaging environment factors such as temperature, pH, gas composition, rotten metabolites, etc., providing consumers with chemical, physical, microbiological, and other quality information [11]. It has the ability to detect and record variations in the food or packaging environment, in order to display food quality information to consumers [12]. There are three main types of intelligent packaging systems for food packaging. Data carriers such as barcodes and radio frequency identification tags (RFID) can be used for warehouse management and product traceability. Sensors such as electronic noses and electronic tongues provide fast and unambiguous quantification of analytes in food using multi-sensor arrays to sense the characteristic response signals of food. Indicators are designed to provide greater convenience and to inform consumers about food quality [13,14].

Regarding current intelligent food packaging materials, indicators such as freshness indicators, gas indicators, and time–temperature indicators have been widely studied, due to their ability to provide qualitative or semi-quantitative information on food through color variations. Among these, intelligent packaging systems based on freshness indicators provide consumers with real-time quality monitoring information on packaged food through quality sensors/indicators, especially the widely developed pH-responsive freshness indicators [1,15,16]. A pH-responsive freshness indicator works by using the following mechanism. The nutrients in food (protein, fat, etc.) decompose under the action of enzymes and microorganisms and produce various acidic and alkaline gases (trimethylamine (TMA), dimethylamine (DMA), ammonia (NH_3_), hydrogen sulfide, carbon dioxide, etc.), which are then released into the confined space during the process of food freshness change [17]. As the storage period increases, these released acidic and basic compounds become denser in the package headspace and are further adsorbed by the freshness indicator attached to the inside of the package. These compounds lead to an increase in hydroxyl ions in the indicator. Accordingly, the deprotonation of hydroxyl groups changes the structure of the natural dyes, which then exhibit color changes. The consumer is made aware of the change in the freshness or quality of the food through the color change of the indicator.

This review introduces current methods for detecting food freshness. The composition and preparation methods of pH-responsive freshness indicators are summarized. Their application in monitoring food freshness and quality is introduced in detail. Mechanistically, the pH properties of foods change as a result of processes such as microbial growth releasing carbon dioxide or carbon dioxide being released on exposure to air. Quite a few commercial indicator systems are already marketed for detecting pH changes as a result of changes in freshness, and this review is strengthened by tabulating and discussing them, in order to identify what further improvements might be possible using natural colorants. Finally, this review covers the application prospects and challenges of pH-responsive freshness indicators based on natural food colorants.

Current pH-responsive freshness indicators usually consist of two main parts: a carrier and the pH-responsive colorants [18]. Indicators are mainly prepared using the following methods: (1) immobilizing the colorants by adsorption on a polymer support, (2) fixing the colorants by covalent interaction with hydrophilic cellulose, or (3) immobilizing the colorants via hydrogen bonding or ionic interaction with the polymer support [19]. Currently, most pH-responsive freshness indicators are prepared with different types of synthetic colorants (including cresol red, methyl red, bromocresol purple, bromocresol green, chlorophenol, bromothymol blue, and xylenol) [20]. However, most synthetic colorants are toxic, and some of them are even carcinogenic, which may pose a serious threat to life and the environment [21]. Hence, it is crucial to find non-toxic and safe colorants for the preparation of pH-responsive freshness indicators. The application of natural colorants could improve food safety, while also meeting consumer expectations for food safety. Recently, natural dyes such as curcumin, alizarin, shikonin, and anthocyanin have been widely used in intelligent packaging research [22,23,24]. Curcumin is a common yellow dye extracted from the roots of curcuma plants [25]. Shikonin and alizarin are red dyes extracted from *Boracaceae* and *Rubiaceae*, respectively [26]. Anthocyanins have been widely studied due to their noticeable and rich color variations over a wide range of changes in pH, high safety, easy availability, and other functional properties such as antioxidant and antibacterial activity [27]. Most natural pigments can be obtained from agricultural waste and food processing plant wastes such as fruit peel, pressed residue, etc. Consequently, research into pH-responsive freshness indicators not only promotes the rational utilization of waste but also provides a green substitute for synthetic dyes for monitoring food quality.

## 2. Methods for Detecting Food Freshness

Detection methods for food freshness cover conventional methods (such as microbiological methods, physical and chemical detection methods, and sensory evaluation methods) and rapid nondestructive testing technology [28]. Conventional detection methods are time-consuming, destructive, and costly [26]. Most of the freshness indicators in the freshness monitoring process need to be evaluated in conjunction with the indicators for the food-quality change process (e.g., total bacterial colony, TVBN value, weight loss, etc.) to further improve the application of freshness indicators [29].

**Figure 1:**
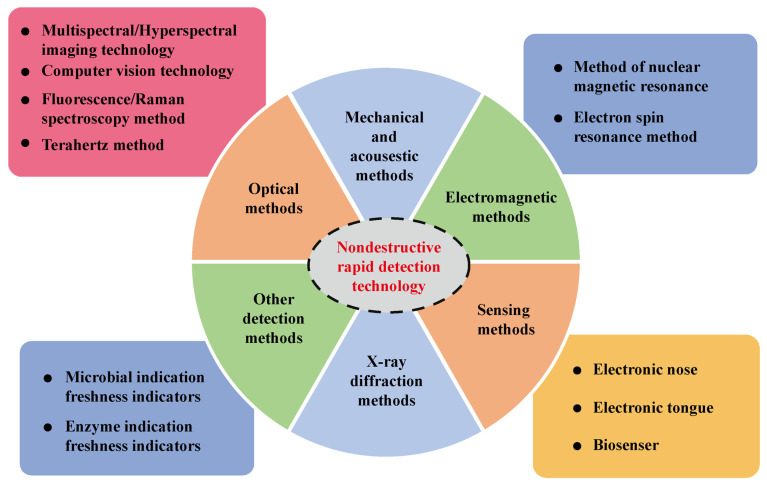
Classification scheme for nondestructive rapid detection techniques in food analysis.

These methods refer to the detection of the internal and external properties, state, and structure of the object by means of physical methods without destroying the object. Traditional chemical detection methods generally require destructive pretreatment of analytes. Unlike traditional detection methods, nondestructive rapid detection techniques possess the advantages of not damaging the samples, fast detection speed, less contamination, and low analysis cost [30]. In food analysis and detection, nondestructive rapid detection techniques can be divided into optical methods, mechanical and acoustic methods, X-ray methods, electromagnetic methods, sensor methods, and other detection methods (microbial indication and enzyme indication freshness indicators), depending on the detection mechanism. Figure 1 shows the specific classification scheme. Electronic nose and tongue technology, biosensors, and pigment sensors have important applications in the identification of the quality of food products [31].

In food analysis, visible/near infrared spectroscopy is mainly used to analyze the composition and quality of food products. Zhang et al. (2010) [32] used headspace solid-phase microextraction (HSSPME) and gas chromatography–mass spectrometry (GC-MS) to investigate the volatile substances in different seafood products at different storage stages. Afterwards, the characteristics of the volatile substances were statistically interpreted using methods such as normalization and principal component analysis (PCA), thus establishing a systematic method for evaluating the freshness of stored seafood. Computer vision technology is a technology that can objectively obtain image information from the food being inspected through optical imaging sensors and then mine the information on food-quality characteristics contained in the image through image processing technology [33]. Xu et al. [33] used a PLS (partial least squares) algorithm to obtain data and developed a quality testing model based on the minimum error probability. The detection accuracy of the method was over 93%, and the classification efficiency was 5400/h, which indicated that the method was feasible for grading salted eggs. Meanwhile, electronic nose and electronic tongue technology has also been gradually applied to the quality identification of food. Han et al. [34] developed a new method for nondestructive detection of fish freshness using electronic nose and electronic tongue techniques combined with chemometric methods. A three-layer radial basis function neural network model was developed for the qualitative discrimination of fish freshness via PCA analysis of the electronic nose and electronic tongue data.

Nondestructive detection techniques have attracted considerable attention, as they are nondestructive with respect to samples and environmentally friendly, with no contamination and a fast detection speed. With the improvement of requirements for food safety, nondestructive detection techniques have been developed in the directions of simplicity, miniaturization, portability, specificity, and high sensitivity. Hence, pH-responsive freshness indicators based on natural food colorants represent an indispensable development direction for non-destructive detection techniques.

## 3. Overview of pH-Responsive Freshness Indicators

Color, pH, and smell, along with variations in the internal and external properties of packaged food, are all important signs of food spoilage. These changes are mainly related to the decomposition of proteins, fats, and sugars by microorganisms and endogenous enzymes, as well as being related to the type of food (fish, meat, milk, vegetables, fruits, etc.) and the storage conditions [35]. In the process of growth and reproduction, microorganisms release various metabolites (hydrogen sulfide, amines, carbon dioxide, water, etc.), resulting in changes in the acidity or alkalinity of the food and the environment around the food. For this reason, pH-sensitive colorants have been introduced into the preparation of freshness indicators. During changes in packaged food freshness, the pH-responsive freshness indicator reacts with the metabolites of microorganisms on the food and shows a color change due to the change in pH value [36,37]. Hence, pH-responsive freshness indicators convert the detection of qualitative or quantitative changes in the concentration of one or more substances associated with food spoilage in a package into a perceptible signal that can be visually detected by the consumer [38]. Color changes in pH indicators are usually attributed to the protonation or deprotonation of carboxylic acid functional groups or amidocyanogen. Additionally, previous research has found that the probability of dissociation occurring within two pH units is about 20% to 80% when only one type of dissociable group exists [39]. Hence, the color range of the freshness indicator is mainly affected by the number of dissociable groups.

Water-vapor permeability (WVP) and oxygen permeability (OP) are key barrier parameters in evaluating polymeric packaging films for food protection and shelf-life extension. Most of the prepared pH-responsive freshness indicators are composed of hydrophilic natural colorants and bio-based materials [40]. They contain more hydrophilic groups (e.g., hydroxyl, carboxyl, etc.), resulting in poor water-vapor permeability of the membrane. Numerous studies have found that electrostatic interactions between substrates in films and cross-linking between natural colorants and film substrates reduce the availability of hydrophilic groups in the substrate, thereby reducing the affinity for water molecules [41,42]. However, pH-responsive freshness indicators still have higher oxygen/moisture permeability than commercial films such as LDPE, polypropylene, and polyvinyl chloride. Hence, future studies may consider the use of multilayer films or the addition of other materials to further reduce the WVP of the freshness indicator.

### 3.1. Synthetic Colorants

As one of the most striking features of food, color is one of the most important standards for recognizing and perceiving the quality and appearance of food, and it directly affects the choices, acceptance and consumption tendencies of consumers [43]. Synthetic colorants are often used as additives in the food industry, especially in confectionery and indicators/sensors, due to their advantages of good stability, excellent color changes, and low cost. Synthetic colorants containing phenolphthalein, bromocresol purple, bromocresol green, and methyl orange are widely applied in pH-responsive freshness indicators, showing red, blue, purple, yellow/orange, green, and other colors in different pH environments [44,45,46]. The prepared freshness indicator presents a reversible color change in contact with gases, liquids, and semisolids with different pH values [47]. Chemical barcode sensors can be prepared by using bromocresol green as a pH-sensitive pigment. By studying the characteristics of the sensor and its response in standard ammonia, it was found that these sensors can show a visible color response to volatile nitrogen compounds [48]. Researchers have conducted sufficient tests on cod and other fish to confirm that the sensor response is related to the growth pattern of bacteria in aquatic products and seafoods, thus enabling real-time monitoring of changes in the freshness of various perishable products. Additionally, other researchers have reported studies with similar principles using sensors prepared from synthetic colorants in intelligent packaging to monitor food freshness [49,50,51]. To establish a more accurate freshness monitoring index system, an indicator based on mixed pH-sensitive pigments was also proposed as a “chemical barcode”, to monitor deterioration in desserts and skinless chicken breasts [52,53]. Furthermore, pH-responsive freshness indicators consisting of two or more pH-sensitive pigments such as bromocresol green and methyl red or bromothymol blue and methyl orange remain in the form of a single sensor. To overcome the disadvantage that it is difficult for a single indicator to accurately monitor food freshness, Kuswandi et al. [54] proposed a package sensor label where dual synthetic dyes were used to prepare dual indicators to detect meat freshness. Since the freshness indicator used two synthetic dyes, the cross-referencing of colors could effectively avoid false positives in freshness testing, resulting in more accurate freshness testing. They found a good correlation between the color variation of the freshness indicator and the sensory evaluation, TVBN, and bacterial growth for packaged beef by applying a dual-dye freshness indicator to the monitoring of beef freshness. The freshness indicator was successfully applied to monitor the real-time freshness of beef at indoor and refrigerated temperatures. Hu et al. [55] prepared a pH-responsive antibacterial film by adding aminoethyl-phloretin to a mixture of polyvinyl alcohol and polyacrylic acid for smart food packaging. The film has strong antibacterial activity against Listeria monocytogenes and Staphylococcus aureus, so it can not only monitor the freshness of pork but also prolong its storage time. In general, synthetic pigments are considered to be teratogenic, carcinogenic, or mutagenic compounds that might pose a potential risk to humans and other organisms, endangering the environment [56]. Consequently, current food research trends and consumers are more inclined towards natural food colorants. The advantages of natural colorants, i.e., vivid colors, nontoxicity, environmental friendliness, and versatility, have encouraged researchers to utilize them as pH-sensitive colorants for freshness indicators [57].

### 3.2. Natural Colorants

Natural colorants have gradually become substitutes for synthetic colorants because they are generally non-toxic or have low toxicity, and they are environmentally friendly and easy to extract. Most natural colorants are polyphenolic compounds, which are widely found in the roots, stems, leaves, and fruits of plants. Natural colorants could be classified into chromogenic groups with conjugated systems (carotenoids, carotenoids, and betaines) and metal-ligated porphyrins (chlorophyll, myoglobin, and their derivatives) according to the chemical structure of their chromogenic groups [58]. Natural colorants are usually extracted by solution extraction, while organic solvents, water, and low-carbon alcohols are used for the extraction of lipophilic colorants and water-soluble colorants, respectively [59,60]. New technologies such as ultrasound, microwaves, and pulsed electric fields could also be applied to extract natural colorants [61]. Natural colorants are used in the preparation of sensors and intelligent packaging systems as well as being used as food colorants, and they can exhibit special color changes in response to acid–base variations in the surrounding environment [62]. The mechanism of pH-responsive freshness indicators based on natural colorants is dependent on the protonation/deprotonation tendency of colorants in acidic/alkaline environments [63]. However, the quality of food could be monitored by other types of sensors that show color changes for specific gases in food [64]. The main colorants used in the preparation of pH-responsive freshness indicators are anthocyanin, curcumin, alizarin, and betalain. Among these, anthocyanins have been widely studied due to their wide color range, easy availability, and fast response speed [65,66].

#### 3.2.1. Anthocyanin

**Figure 2:**
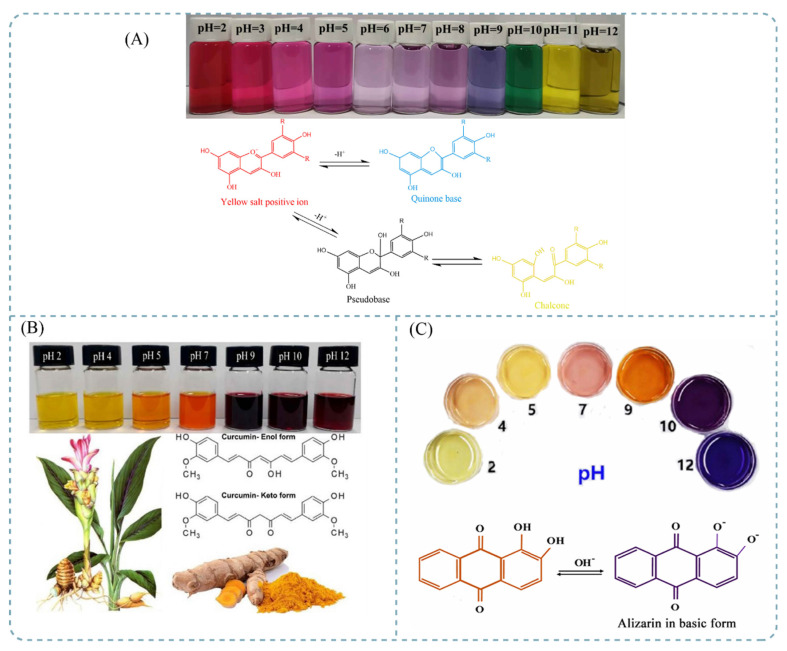
Color changes and mechanisms for: (**A**) anthocyanins [5]; (**B**) curcumin [76]; (**C**) alizarin at different pH values [77]. Source: reprinted with permission from Liu et al. [5], 2021, Elsevier; Ezati et al. [76], 2020, Elsevier; and Roy et al. [77], 2021, ACS.

Anthocyanins are flavonoid compounds formed from glycosylated polyhydroxy or polymethoxy derivatives of 2-phenylchromen, a natural colorant that reflects light from red to blue in the visible spectrum [67]. More than 600 types of anthocyanins have been found in the environment. There are mainly six types of anthocyanins in plants: cyanidin, petunidin, delphinidin, malvidin, peonidin, and pelargonidin [68,69]. The color-changing function of anthocyanins as pH-sensitive colorants depends on the structural changes caused by the acid–base properties of the environment [70]. When the environment is strongly acidic, the structure of anthocyanin is mainly in the form of red flavylium cations [71]. With an increase in pH value, the flavylium cation structure is destroyed and rapidly hydrated on C-2 to form a colorless carbinol pseudobase, and the red color becomes pale. Under neutral and alkaline conditions, a large number of purple quinone base structures and blue quinone base structures are produced and gradually transformed into pale-yellow chalcone structures [72]. The stability and color of anthocyanin is greatly influenced by external conditions such as pH value, temperature, enzymes, metal ions, and so on, which have influences on the application as a freshness indicator [73]. A large number of studies have indicated that the interaction between anthocyanins and bio-based materials plays an important role in enhancing the stability of anthocyanins, which may be controlled by electrostatic interactions [74]. Other studies have proved that intermolecular forces can further extend the π–π conjugate system of anthocyanins and further enhance their color-changing effect [75]. The color changes and mechanism for anthocyanins are shown in Figure 2A.

#### 3.2.2. Curcumin

Curcumin is a diketone compound extracted from the rhizome of zingiberaceae, which has excellent anticancer and anti-inflammatory effects. The main curcumin compounds in turmeric are curcumin, demethoxycurcumin, didemethoxycurcumin, and cyclocurcumin [78]. Curcumin has been recognized as a powerful antioxidant due to the presence of an O-methoxyl group, and its multi-dimensional therapeutic effect on numerous chronic diseases has been well proved. It can be used in the preparation of food packaging films on account of its antibacterial and antioxidant properties [76]. Meanwhile, due to its structure change under different pH conditions, curcumin can present visible color variations in different acid–base environments. Curcumin possesses an ordered crystal structure composed of β-diketone groups consisting of two aromatic rings with a methoxyl group and a phenolic hydroxyl group. Enol and keto are two possible tautomeric forms of curcumin, which can be transformed by pH changes in the environment. In polar, acidic, and neutral media, the keto form is dominant, while in non-polar and alkaline environments, the enol forms appear [79]. The structural features of curcumin are illustrated in Figure 2B. Curcumin usually has poor solubility in aqueous solutions, and its stability decreases under strong acid and alkaline conditions [80]. Hence, the extraction method for curcumin mainly uses organic solvents for liquid extraction [81]. Research on freshness indicators based on curcumin remains surprisingly scarce due to the poor stability and solubility of curcumin in strong acid and alkali environments.

#### 3.2.3. Alizarin

Alizarin (1,2-dihydroxyanthraquinone, C_14_H_8_O_4_) is an orange crystal dye derived from the root of Rubia officinalis. It is often used as a fabric colorant in industry [82]. Via the transfer of protons, the hydroxyl group of alizarin interacts with the carbonyl group to form hydrogen bonds, with a color change from yellow to purple [83]. In the case of low pH values, the remaining charged molecules appear yellow due to ionization of the phenolic hydroxyl groups on alizarin and the effect of the azo groups in azobenzene dyes. With an increase in pH value, primary and secondary dissociations of phenolic hydroxyl groups occur under the action of a resonance effect, leading to the appearance and accumulation of single anions in the solution; thus, the solution changes from yellow to purple [77]. Additionally, alizarin also possesses anti-ultraviolet and antibacterial properties. Since microbial growth and enzymatic decomposition release volatile alkaline compounds to change the pH of food, the pH-responsive discoloration of alizarin can be used as a freshness indicator for packaged food [84]. Alizarin has been used in the preparation of meat freshness indicators. Figure 2C shows the color change and mechanism for alizarin.

#### 3.2.4. Betalain

Betalains are water-soluble colorants found in amaranth, beets, prickly ash, and dragon fruit plants [85]. Currently, they are used as food additives in various foods such as meat, dairy products, poultry, soft drinks, and so on. Structurally, betalains can be classified into yellow/orange betacyanins and red/purple betacyanins [86]. Red/purple betacyanins are composed of cyclo-3,4-dihydroxyphenylalanine (cyclodopa) and beet acid (chromophore). Yellow/orange betacyanins are condensation products of beet acid with an amino acid or amine [87]. The structure of betalain is relatively stable in neutral and acidic environments, so it is often used as an additive in acidic food. The structure and color of betalain changes with an increase in environmental alkalinity and with changes in temperature and light. In strong alkali solutions, betalains can gradually degrade into colorless cyclo-DOPA 5-O-(malonyl)-β-glucoside and yellow betaxanthins [88]. Recently, a number of experiments have proved that betalains possess antibacterial, anticancer, lipid-lowering, and antidiabetic properties [89]. They can be used in the preparation of active packaging and intelligent packaging due to their antibacterial, antioxidant, and pH-dependent color-changing properties. Betalains are polyfunctional pigments that can be used in the preparation of smart indicators due to their color diversity in different acid–base environments.

A large number of studies have revealed that natural colorants can be used in monitoring food freshness. Natural colorants are generally non-toxic or have low toxicity, with antioxidant and antibacterial properties that contribute to prolonging the quality guarantee period of food in active packaging. However, natural colorants possess certain limitations, including strong water solubility and degradation under hard light, harsh temperatures, and non-neutral conditions. Consequently, the future development of natural colorants should overcome their shortcomings, in order to further expand their scope of application.

#### 3.2.5. Shikonin

Comfrey is a herb with a wide range of pharmacological activities, including wound-healing, antibacterial, anti-inflammatory, and antitumor activities. Shikonin is a natural colorant extracted from comfrey. Its main chain consists of alternating 1,3-linked-D-galactose and 1,4-linked 3,6-anhydro-L-galactose units, with a complex multiphase structure [90]. Anthraquinone pigment is also a pH-sensitive dye with a more stable structure than the hydrophilic anthocyanin. In addition, the structure of shikonin contains more hydrophobic groups, so its application in the preparation of pH-responsive freshness indicators can enhance the hydrophobicity of the indicator and thus expand its range of application. Huang et al. [91] incorporated shikonin into agar for the preparation of freshness indicator films and applied it to the freshness monitoring of fish. It was found that the color response of the freshness label was consistent with the deterioration threshold of the total viable count (TVC) and total volatile basic nitrogen (TVBN) content in fish samples. The indicator film provided a nondestructive and convenient way to assess the freshness of fish in storage. Dong et al. [90] prepared a novel hydrophobic colorimetric film using cellulose and shikonin to improve the mechanical properties and hydrophobicity of the colorimetric sensing membrane. The colorimetric film could monitor the freshness of shrimp and pork under storage conditions of 20 °C, 4 °C, and −20 °C. Further findings revealed that the performance of the novel colorimetric film for freshness monitoring of meat products was consistent with the current Chinese standard. Huang and Dong also investigated the stability of shikonin and found a slight red shift in its UV spectrum under acidic conditions, which proved that its structure was more stable under acidic conditions [90,91]. However, the absorption peak intensity in the UV spectrum increased with an increase in alkalinity, indicating that the chromophore molecular structure in shikonin changes under alkaline conditions.

### 3.3. Polymer Support

Generally, the polymer support used to immobilize pigments is a key component of pH-responsive freshness indicators. As an important component of pH-responsive freshness indicators, polymer supports can be divided into synthetic polymers and biopolymers (proteins and polysaccharides). It is important to note that the polymer support must meet some basic requirements for the preparation of pH-responsive freshness indicators. The polymer support must have the following properties. (1) The polymer should be a water-based polymer, which is helpful for the fixation of water-soluble natural pigments. (2) The polymer should be almost colorless to avoid masking the color of the natural dyes and affecting the monitoring. (3) The polymer should ensure the stability of natural pigments at low or high pH values. (4) The polymer should possess sufficient mechanical strength. Numerous synthetic polymers and biopolymers have been used in the preparation of freshness indicators, e.g., filter paper, polyethylene, starch, polyvinyl alcohol, chitosan, cellulose, and κ-carrageenan [92,93].

#### 3.3.1. Synthetic Polymers

Synthetic polymer supports are mainly based on petroleum polymer, which possesses excellent physical, chemical, and mechanical properties for resisting external temperature, microbial, and physical/chemical damage. Pacquit et al. [48] first obtained a smart sensor by coating a polyethylene terephthalate (PET) film with bromocresol green. The smart sensor detects changes in fish freshness quickly and shows great potential as a food-quality indicator. Since the dye migrates during application and its response to pH changes is susceptible to temperature, Kuswandi et al. (2012) [94] developed a novel colorimetric method based on polyaniline (PANI) films that could be used to monitor changes in fish freshness in real time, while being reusable after acid solution treatment. Wang et al. (2018) [95] prepared a functional reproducible colorimetric indicator based on polyaniline that could be used for fish freshness monitoring. Due to the toxic and carcinogenic problems of synthetic dyes, Zhai et al. (2020) [96] prepared a non-toxic, low-cost colorimetric gas sensor using curcumin, a natural dye, co-extruded with low-density polyethylene (LDPE). With good stability and accurate monitoring of the TVBN gas associated with meat spoilage, the sensor has good prospects for smart packaging applications. Although petroleum-based polymer products are convenient, there are numerous environmental and health issues associated with them, so the search for suitable green materials to replace petroleum-based polymers is urgent.

#### 3.3.2. Biopolymers

Recently, biopolymers have gained widespread interest due to their biodegradability and the accumulation of petroleum-based packaging polymers in the environment. The synthetic polymer support is mainly based on a petroleum polymer, which possesses excellent physical, chemical, and mechanical properties for resisting external temperature, microbial, and physical/chemical damage. The widespread application of petroleum-based polymers and the lack of degradation have a huge harmful impact on the environment. Consumers are increasingly inclined to choose environmentally friendly polymers, due to the implementation of global government environmental policies and increased consumer awareness of environmental protection. There are many materials on the market to replace petroleum-based polymers. The application of renewable resources to developing environmentally friendly and biodegradable materials could ameliorate the health and environmental problems associated with the application of petroleum-based materials in food packaging [97].

Biopolymers are natural polymers derived from living organisms. They contain monomer units with covalent bonds that degrade naturally in the environment [98]. Biopolymers such as lipids, polysaccharides, and proteins have been used in the preparation of packaging films. Polysaccharide biopolymers can form hydrogen bond and ion interactions with pH-responsive colorants to enhance their color-changing effect and reduce the interference of the external environment. Hence, biopolymers such as chitosan, sodium alginate, cellulose, and pectin have been widely used in the preparation of pH-responsive freshness indicators [99,100]. Additionally, fruit and vegetable processing wastes such as fruit, peel, and residue could become a rich source of cellulose and polysaccharide biopolymers [101]. Biopolymers are widely used in active packaging and intelligent packaging due to their non-biotoxicity, biodegradability, and excellent compatibility. Biopolymers are often mixed with other polymers or modified materials to improve their water-solubility and poor mechanical and barrier properties.

## 4. Preparation of pH-Responsive Freshness Indicators

**Figure 3:**
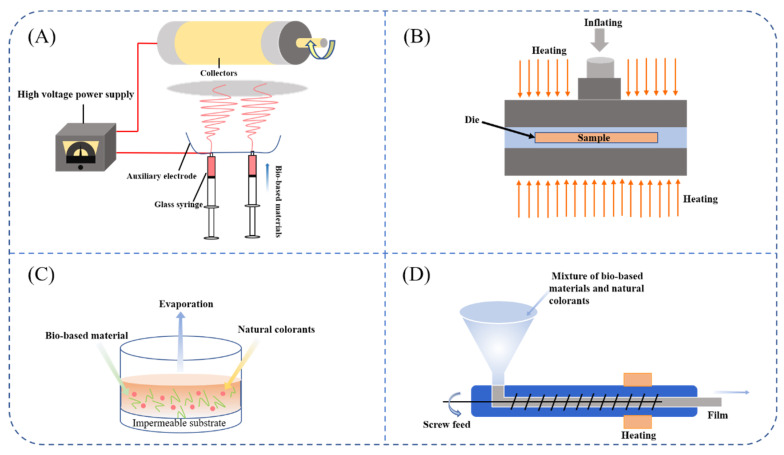
The preparation methods for freshness indicators: (**A**) electrospinning; (**B**) compression molding; (**C**) solvent casting; (**D**) extrusion.

The preparation methods for pH-responsive freshness indicators include casting, blending extrusion, compression molding, electrostatic spinning, coating, adsorption, and electrochemical etching. Figure 3 shows the process for preparing freshness indicators. The process of freshness indicator formation is influenced by several factors such as the molecular structure and compatibility of biopolymers and the particular application. Different preparation methods have an influence on the monitoring effect and stability of the freshness indicator. Hence, in this section, we discuss the preparation methods and deficiencies of pH-responsive freshness indicators.

### 4.1. Freshness Indicator Preparation by Solvent Casting

Solvent casting or flow drying is a common method of preparing freshness indicators at laboratory or pilot scales. The preparation for casting is mainly divided into the following three processes: dissolution, casting, and drying/molding. Firstly, the biopolymer, additives, and other components are dissolved in a suitable solvent to develop the mixed solution. The preparation conditions of the mixed solution mainly depend on the structure and application scenarios of the material. Then, the mixed solution is cast in a specific mold. The final step is drying, which promotes interactions among the polymeric molecules. It is a necessary step for obtaining excellent performance from the freshness indicator. Drying temperatures usually range from 20 °C to 60 °C. The drying temperature determines the drying time, which usually ranges from 6 h to 3 days.

Biodegradable materials such as polyvinyl alcohol, polysaccharide (cellulose, glue, chitosan, sodium alginate, etc.), and protein are often used in the preparation of freshness indicators. The hydrogen bonds between the polysaccharide polymer and the natural colorants can further promote their interaction, stabilize the natural colorants, and improve the effect of the color change in the slow casting process [102]. Compared with other polysaccharides or phenols, pectin and natural colorants have the highest affinity, which might be affected by electrostatic interaction and anthocyanin accumulation [103]. Accordingly, most of the studies on freshness indicators are related to pectin [77]. It is found that hydrophilic materials help the freshness indicator to absorb more water in the application process and react directly with volatile basic nitrogen to form NH4^+^, thus accelerating its color variation [104] (Table 1).

### 4.2. Freshness Indicator Preparation by Extrusion

The disadvantage of preparing freshness indicators by casting is that the evaporation process of the solution cannot be controlled, which leads to a residue of toxic substances in the freshness indicator, harming human health in the process of application [96]. The mechanical properties and barrier properties of freshness indicators prepared by the casting method are also poor.

Extrusion has become one of the main processing methods for petroleum-based polymers, including ethylene vinyl alcohol copolymer (EVOH) and polypropylene (PP). The working temperature for extrusion molding is generally 180 °C–290 °C, while the processing temperature of petroleum-based materials is above 200 °C, due to their excellent thermal stability. The polymer material is easily degraded by moisture in the processing; the water content of the film matrix is required to be very low. The extrusion process is mainly composed of the following three parts: (1) the feeding zone, where the polymer is evenly mixed under pressure and the action of the screw and moved into the next region, (2) the kneading zone, where the mixture is further homogenized by removing air under the action of a screw and high temperature, and (3) the equalization zone, where the mixture, which is a molten viscous fluid in this area, is extruded quantitatively from the machine head [110].

Mills et al. [105] first proposed preparing a hydrophobic gas-sensitive film by embedding colorants in polymer plastic by extrusion (Table 1). The main operation steps included feeding, melt plasticizing, extruding the film tube, blowing, shaping, and so on. With regard to hydrophobic polymers, extrusion possesses numerous advantages such as fast preparation speed, ease of control, and high production. Bromophenol blue was embedded in low-density polyethylene (LDPE) by extrusion to prepare a hydrophobic NH_3_-sensitive film which was successfully applied in smart packaging to monitor the real-time freshness of fish [64]. Zhai et al. [96] prepared a hydrophobic biogenic amine-sensitive film by encapsulating curcumin in LDPE using a melt extrusion blowing method, with curcumin as an indicator (Table 1). The film exhibited excellent mechanical properties and barrier properties, together with the potential for application in food freshness monitoring.

### 4.3. Freshness Indicator Preparation by Electrospinning

The high temperatures and pressures of extrusion can affect and deactivate the colorants in the freshness indicator. Electrospinning is an effective and versatile method for the preparation of nonwoven and continuous polymer nanofibers, with additional advantages with respect to orientation, excellent porosity, and fiber uniformity. Thanks to these splendid and interesting properties, electrospun nanofibers have been used in the preparation of food packaging materials [111]. In the process of electrospinning, the polymer solution or melt at the tip of the needle changes from a sphere to a cone (Taylor cone) and extends from the tip of the cone, giving fiber filaments under the action of a strong electric field. Generally, a high-voltage electric field, a nozzle, and a metal collection plate are the crucial components of the electrostatic spinning process. Specifically, the spun yarn ejected from the spinning needle is drawn and split by the external constant high-voltage electric field, while the solvent in the spun yarn evaporates rapidly. Due to electrostatic repulsion and stretching coupled with solvent volatilization, the fluid forms fibers with a small diameter. After curing, the fibers are arranged in a disordered fashion on the collection device to form a fiber felt similar to nonwoven fabrics [112]. The spinning voltage, polymer solution concentration, solvent volatility, and extrusion speed are the main factors affecting the performance of electrostatically spun fibers.

According to the different forms of raw materials, electrospinning technology can be classified into solution electrospinning and melt electrospinning [113,114]. It can also be split into needleless electrostatic spinning, coaxial or triaxial electrostatic spinning, and multi-jet electrostatic spinning, according to the design of the nozzle [115,116]. Electrostatic spinning technology is mostly applied to food preservation and antibacterial packaging and is less used in the study of freshness indicators. Liu et al. [117] prepared films using curcumin-containing maize alcoholic protein by forming fibers using electrostatic spinning. The films exhibited excellent antibacterial and antioxidant activities. Yildiz et al. [106] prepared a pH-halochromic sensor based on electrospinning nanofibers, utilizing curcumin, chitosan (CS), and polyethylene oxide (PEO) for detecting chicken freshness (Table 1). Their experimental results showed that curcumin nanofibers met the application expectations for providing visualization for detecting chicken spoilage.

### 4.4. Freshness Indicator Preparation by Compression Molding

Compression molding is a common method used in polymer processing to prepare continuous polymer materials by melting the polymer matrix at high temperature and pressure. Additionally, compression molding is a simple, fast, and low-cost method. Recently, Uranga et al. [107] prepared bio-based films based on anthocyanin and fish gelatin from food processing waste using compression molding and used them for active packaging (Table 1). Using compression molding, it is easier and faster to produce films in large quantities. Further investigation showed that the films prepared by the molding process were homogeneous. The addition of anthocyanins changed the optical properties of the film, making the film surface rougher, but improved its antioxidant properties, suggesting that the colorimetric film could be used as an active film to extend the shelf life of food products. In another study, Andretta et al. [62] prepared starch-based films by compression molding using blueberry pomace as a pH-sensitive colorant. They found that the addition of blueberry residues resulted in poor uniformity of the starch-based film but had no significant effect on the water content, moisture permeability, or mechanical properties of the film. Colorimetric analysis of the film revealed that it exhibited visually perceptible color changes in buffers with different pH values, indicating the potential for application in smart packaging. However, anthocyanins can be degraded when they are exposed to high temperatures during compression molding and used in food packaging applications, due to interactions with visible light. Gaviria et al. [108] developed pH-indicator films based on cassava starch, Laponite, and jambolan (*Syzygium cumini*) fruit using compression molding (Table 1). Due to the presence of colorants such as anthocyanins in jambolan fruit, the film appeared purple. The addition of Laponite and jambolan fruit affected the chemical structure, the crystallinity, and the distance between the starch molecular chains of the starch-based film. The application of monitoring steak freshness at different temperatures showed that the film could exhibit significant color changes. Therefore, compression molding can be used for the mass production of freshness indicators by changing the working conditions of the compression molding process and adding other protective materials to the indicator preparation.

### 4.5. Freshness Indicator Preparation by Other Methods

Most of the early freshness indicators were prepared using synthetic dyes and filter paper, and the dyes were fixed on the filter paper by adsorption [118]. Nevertheless, the freshness indicators prepared in this way were poorly monitored and prone to dye migration, threatening human health. Printing was used to improve the preparation of freshness indicators in a follow-up study. Three-dimensional (3D) printing technology has been widely used in the packaging, food, medical, and other industries due to the advantages of time-saving processes, good shapes, and convenient operation [119]. Based on the rapid development of 3D printing and food packaging, some researchers have proposed that 4D printing could offer a response over time to different environmental factors. A recent study reported the utilization of mashed potatoes and anthocyanins extracted from purple sweet potatoes to prepare a sample that could change color in different acidic and alkaline environments, using a 4D printing method [109] (Table 1). Consequently, natural colorants might be promising raw materials for 4D printing of food packaging in the future, due to spontaneous color changes under pH-stimulating conditions. To date, there has been little work focused on 4D printing for smart food packaging.

## 5. Application of Natural-Colorants-Based pH-Responsive Freshness Indicators

Evaluation of food quality is commonly achieved by destructive and time-consuming methods such as chemical and microbiological methods, which are usually used in the laboratory and require a great deal of time for analyzing the results. Changes in food quality begin initially and continue to occur in transportation, storage, and distribution. The pH-responsive freshness indicator, together with the properties of nondestructive packaging, could allow the freshness of the food to be presented in real time. Table 2 shows the application of pH-responsive freshness indicators in different food-quality monitoring applications.

The application of pH-responsive freshness indicators for food freshness monitoring has shown that their color changes as the food deteriorates. Nevertheless, the effectiveness of different pH-responsive freshness indicators for food freshness detection is directly related to the source and concentration of natural colorants, the carrier matrix, and the preparation method [132]. Researchers should design and develop indicators suitable for monitoring the freshness of food based on the freshness variation characteristics. Table 3 presents examples of color changes in different pH-responsive freshness indicators for different food freshness monitoring applications. Most studies have not only described the relationship between the color variation of the pH-responsive freshness indicator and the microbial spoilage but also linked it to the shelf life of foods. In the case of high-protein foods (e.g., pork, shrimp, fish, beef), researchers have found that these foods should not be consumed when the volatile basic nitrogen value (TVBN) is greater than the current standard value and the microbial count is higher than 7 lg CFU/g [133,134].

### 5.1. Freshness Monitoring of Meat and Seafood Products

Meat and seafood products contain large amounts of protein, fat, and free amino acids, which are susceptible to spoilage and deterioration due to the action of microorganisms and enzymes during storage, transportation, and marketing, resulting in changes in their pH and the production of volatile basic nitrogen (TVBN) species such as ammonia, methylamine, dimethylamine, trimethylamine, and other similar compounds [139]. Among the current smart food packaging materials, indicators (freshness indicators, gas indicators, and time and temperature indicators) have been widely studied for their ability to provide qualitative or semi-quantitative indicators of food properties through color changes. A smart packaging system based on a pH-responsive freshness indicator could provide consumers with quality information on various foods by utilizing a pH-dependent color variation that is perceptible with the naked eye [140,141].

Zhang et al. [66] developed a colorimetric pH-sensing film by immobilizing natural colorants extracted from Chinese redbud flowers with chitosan, which changed color from red to green in different acidic and alkaline environments. They evaluated the response time, stability, and reproducibility with respect to storage time of pH-sensing films. As the pH values of pork or fish samples were related to their freshness, pH-sensing films were identified as a rapid, nondestructive, and intuitive way to estimate the change in pork and fish freshness in different environments. Othman et al. [142] found that the colorants in hibiscus changed color in response to environmental changes in acidity and alkalinity. They developed a pH-based detection system using degradable materials such as chitosan, corn starch, and hibiscus extract to monitor the freshness of chicken breasts. Choi et al. [15] prepared a novel colorimetric film by adding purple sweet potato anthocyanin to a mixture of agar and potato starch agar, and the results indicated that the film could indicate the pH changes and spoilage of pork. Luchese et al. [121] developed biodegradable smart films using blueberry residues, cassava starch, and glycerol as an alternative to petroleum-based plastics. The results of their study indicated that the prepared biodegradable film containing agro-industrial residues had potential as a freshness indicator. A similar study was carried out by Dudnyk et al. [143], who developed pectin films containing red cabbage anthocyanins used for monitoring the freshness of various high-protein perishable foods. Due to the release of volatile amines during microbial growth, the color of the film altered sharply from purple to yellow as the alkalinity of the environment increased. Another study was carried out by Zhang et al. [144], utilizing roselle extracts and biodegradable polymers to prepare an intelligent colorimetric film to detect the freshness of packaged pork. They prepared three types of films, utilizing starch, polyvinyl alcohol, and chitosan. It was found that the film based on polyvinyl alcohol/chitosan/roselle extracts possessed the highest sensitivity to ammonia vapor and could be used as a visual indicator of pork freshness at room temperature (25 °C). The film was initially red, gradually changing to green or yellow over a certain period of time, showing that the freshness of the pork changed over time. Liu et al. [5] developed a novel colorimetric film by mixing sodium carboxymethyl cellulose/ polyvinyl alcohol and red cabbage to detect the freshness of pork. They found that the electrostatic interaction between the mixed film and anthocyanins improved the sensitivity and color stability of the film. The poor mechanical strength and hydrophilicity of smart colorimetric films are the main problems that prevent their large-scale application. Researchers have prepared a pH-sensing film with excellent mechanical properties using biodegradable cellulose and naphthoquinone colorants (AENDs) extracted from comfrey [90]. The colorimetric film has promising applications in preparing smart labels with excellent mechanical properties and hydrophobicity, for freshness detection in shrimp.

To address the problems of the poor hydrophobicity and stability of the freshness indicators and the migration of natural dyes, Zhang et al. [145] designed a novel freshness indicator. The indicator consisted of a sensing layer and a hydrophobic protective layer, which had been verified as capable of monitoring the freshness of food while having good hydrophobicity. Zhang et al. [146] microencapsulated mulberry extracts using a microencapsulation technique, compounding them with psyllium seed gum to prepare pH-responsive films. The films acted as a type of pH-sensitive food packaging material, while ensuring the stability of the natural colorants.

### 5.2. Freshness Monitoring of Milk and Dairy Products

Milk and dairy products are nutrient-rich foods that are highly susceptible to decomposition by microorganisms and enzymes during storage, which can negatively impact their quality attributes and safety [147]. Hence, intelligent packaging is critical in identifying the expiration date and quality of these foods during transportation and consumption. There has been a great deal of research on active packaging for dairy products in the past, but only a few studies have focused on freshness indicators based on natural dyes.

**Figure 4:**
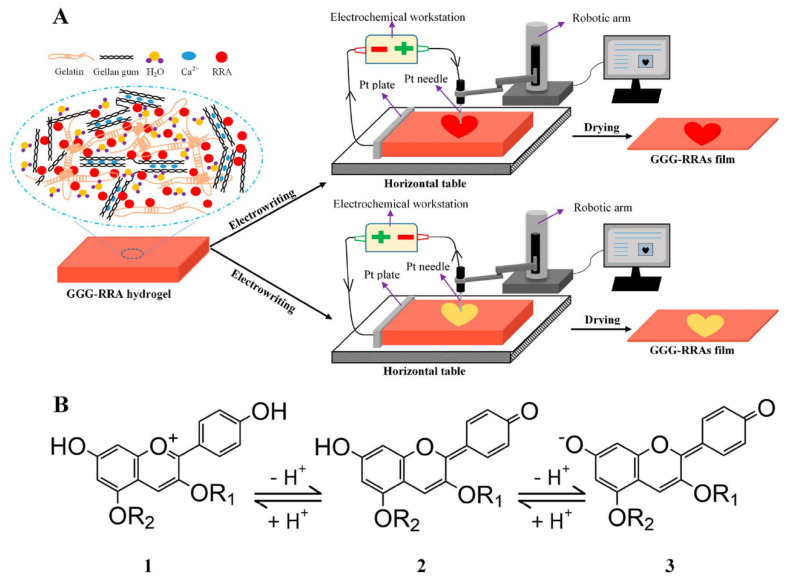
(**A**) Principle of electrochemical writing on pH-sensitive films. (**B**) The structure transformation of red radish anthocyanins in acidic and basic conditions. Source: reprinted with permission from Zhai et al. [150], 2018, ACS.

Stefani et al. [148] prepared a freshness indicator based on polyvinyl alcohol/chitosan incorporating anthocyanins obtained from red cabbage. The color changes of this indicator provided an inexpensive and simple way to present a variation in the chemical composition of the milk. The color of the indicator gradually changed from gray to pink, showing that the milk had deteriorated. In a different experiment, Ma et al. [129] developed colorimetric indicators to detect milk freshness by incorporating grape-skin extracts into a mixture of cellulose nanocrystal (CNC)/tara gum (TG). The color changed from red (acidic environment) to slightly green (alkaline environment) when the indicator was placed in different buffers. The indicator exhibited significant color changes during the variation in milk freshness, indicating that it could be applied for detecting the freshness of dairy products. Liu et al. [149] developed intelligent films based on starch/polyvinyl alcohol (PVA) that monitored pH changes and inhibited the growth of harmful microorganisms in food. Their water resistance and mechanical properties were enhanced by modifying the matrix with sodium trimetaphosphate and boric acid. The films could detect changes in the freshness of milk and prolong its shelf life under the action of anthocyanins (ANT) and limonene (LIM). Zhai et al. [150] also prepared a pH-sensitive film for smart food packaging utilizing gellan gum, gelatin, and carrot extracts as the main raw materials. The composite film presented a color variation from orange to yellow in different pH environments. As shown in Figure 4, the film showed visible color variation, indicating that the food had spoiled during the application of fish freshness detection, while the written pattern was retained. Consequently, the colorimetric film could be used as part of a smart packaging system for monitoring dairy products. In similar research, Yong et al. [29] prepared an active smart packaging film using chitosan and purple or black eggplant extracts (PEE and BEE). PEE and BEE improved the mechanical properties, oxidation resistance, and pH sensitivity of the film. The authors found that an intelligent film with high anthocyanin content could be used to detect milk spoilage, with good application prospects in the field of food freshness detection. Bandyopadhyay et al. [131] developed intelligent films consisting of polyvinyl pyrrolidone-carboxymethyl cellulose-bacterial cellulose-guar gum (PVP-CMC-BC-GG) and anthocyanins derived from red cabbage to detect cheese quality. Initially, the pH-responsive film appeared slightly pink due to the presence of lactic acid and other acids in the cheese. During the freshness monitoring process, the anthocyanins in the responsive film were converted to a reddish yellow molten-salt positive-ion structure due to the production of large amounts of organic acids by microorganisms in the cheese. The films exhibited significant color changes in cheese freshness detection applications.

### 5.3. Freshness Monitoring of Fruits and Vegetables

Fresh-cut fruits and vegetables are highly perishable foods, whose quality and safety may deteriorate during storage due to biochemical processes resulting from pests, microbial contamination, or respiration [151]. Hence, some researchers have developed smart and active packaging materials for quality protection of fresh-cut fruits and for monitoring their freshness changes to reduce food-borne diseases [152]. Chen et al. [153] prepared pH-sensitive labels using a mixture of methyl red and bromocresol blue dyes. During pepper freshness monitoring, the label showed the change in pepper freshness through a visible color change that was due to the increase in CO_2_ concentration as a result of pepper respiration. Consequently, labels made from methyl red and bromothymol blue could be an easy-to-use indicator to detect the freshness of packaged peppers. Smart films made by incorporating anthocyanin-rich blackberry extract into carboxymethyl cellulose (CMC) have also been developed to prolong the shelf life of tomatoes [154]. Overall, there are only a few studies on using natural colorants to prepare pH-responsive films for monitoring the freshness and prolonging the shelf life of fresh-cut vegetables and fruits. Researchers typically spray or macerate fresh fruits and vegetables with a variety of edible materials to create a semi-permeable coating on their surface for controlling populations of natural bacteria, molds, yeasts, and food-borne pathogens [155].

Currently, numerous studies have been conducted on freshness indicators based on various biomaterials and natural pigments, where the color changes of indicators can detect the real-time quality changes in food during storage. In particular, as awareness of environmental protection and food safety increases, the requirement for indicator films utilizing eco-friendly biomaterials and natural pigments to replace petroleum-based materials and synthetic pigments is increasing in the smart packaging sector.

## 6. Conclusions and Future Perspective

This document provided a classification and overview of pH-responsive freshness indicators based on natural colorants. Smart packaging is growing in importance in the production and distribution of food. Smart packaging based on natural colorants can increase the safety and quality of packaged foods by informing consumers about real-time freshness and extending the shelf life of foods.

Nonetheless, pH-responsive freshness indicators based on natural dyes are still a long way from commercial application. In addition, pH-responsive freshness indicators based on natural colorants have obvious disadvantages, for example: (1) natural colorants have lower pH sensitivity than synthetic dyes; (2) natural colorants have poor color stability and good water solubility; (3) the existing process is not suitable for large-scale processing; and (4) there is a low matching degree with food quality indicators in packaging. There are still limitations and gaps in the large-scale use of natural colorants for food freshness indication. To overcome these problems, the following recommendations are proposed: (1) finding natural colorants with more stable performances and excellent heat resistance or improving the thermal stability of natural colorants by encapsulation and immobilization; (2) modifying the carrier matrix to reduce the water solubility of the carrier matrix and improve compatibility with colorants; and (3) improving the pH sensitivity of freshness indicators by adding nanomaterials or other new additives.

Consequently, the following perspectives are available for efficient intelligent packaging based on pH-responsive indicators. (1) A more pH-sensitive freshness indicator should be prepared for detecting subtle changes in freshness, because less alkaline gas is produced at the initial stage of food freshness change. (2) The types and amounts of volatile nitrogenous compounds produced by different foods during changes in freshness vary from food to food. The developed pH-responsive freshness indicators are only suitable for freshness detection in certain types of foods. Hence, the follow-up development of efficient intelligent packaging should be in the direction of convenience, speed, and real-time display of food quality changes, in order to reduce health problems caused by poor food quality and safety.

## Figures and Tables

**Table 1:** Methods of developing pH-responsive freshness indicators.

Materials	Colorants	Methods	Food Sample	Reference
Pectin/Sulfur nanoparticles	Curcumin	Solvent Casting	Shrimp	[77]
Chitosan/Microcrystalline cellulose	Curcumin	Solvent Casting	No date	[104]
Poly (vinyl butyral) or ethylcellulose/Tributyl phosphate	Bromophenol blue, Bromocresol green or Chlorophenol red	Extrusion	No date	[105]
Low-density Polyethylene/SiO_2_ nanoparticles	Bromophenol blue	Extrusion	Fish	[64]
Low-density polyethylene (LDPE)	Curcumin	Extrusion	Silver carp/Beef	[96]
Chitosan/Polyethylene oxide	Curcumin	Electrospinning	Chicken breast	[106]
Fish gelatin	Anthocyanin	Compression Molding	No date	[107]
Cassava starch/Laponite	Anthocyanin	Compression Molding	Round steak	[108]
Potato flakes/Sodium alginate powder/Citric acid	Anthocyanin	4D printing	No date	[109]

**Table 2:** Natural-colorants-based pH-responsive freshness indicators for monitoring freshness of food products.

Tested Food	Natural Colorants	Source of Natural Colorants	Polymer Materials	pH Values/Color Variations	Colorant Concentrations	Reference
Pork and shrimp	Curcumin	Curcuma longa	k-carrageenan	3 and 13 Pink and blue-green colors in different solutions	Ethanol solutions (10 mL, ethanol/water = 4/1, *w*/*w*) containing 0, 1, 3, 5, and 7% (*w*/*w*) based on k-carrageenan	[23]
Pork	Anthocyanin	Prunus maackii	κ-carrageenan/hydroxypropyl methylcellulose	3–11 Dark red to gray-blue colors	0, 2, 4, 8, and 16% (*w*/*w*) based on total mixed hydrogels	[120]
Chicken	Anthocyanin	Blueberry residue	Cassava starch	2–11 Red/pink /purple (pH ≤ 5) and yellowish (pH ≥ 6)	4 g blueberry residue powder/100 g cassava starch	[121]
Chicken/fish	Betalains	Amaranthus leaf	Polyvinyl alcohol/gelatin	2–11 Pink (pH ≤ 4) to bluish pink (pH ≤ 6) to blue (pH ≤ 9) and to gray (pH ≤ 11)	5% Amaranthus leaf extract (*v*/*v*) based on total mixed hydrogels	[122]
Minced beef	Alizarin	Roots of Madder family plants	Cellulose/chitosan	2–11 Yellow to dark purple	1% (*w*/*v*) based on total mixed hydrogels	[16]
Seafood/meat	Alizarin	Roots of Madder family	Carboxymethyl Cellulose/Agar	2–12 Yellow at acidic pHs, pale pink at neutral pH, and red to purple to blue at basic pHs	1.0% (*w*/*w*) based on mixed hydrogels	[83]
Fish	Anthocyanin	Black carrot	Bacterial nanocellulose	2–11 Red to khaki	6 mg/mL	[70]
Fish (Bighead carp)	Curcumin (CR)/anthocyanin (ATH)	Curcuma longa/Purple sweet potatoes	Starch/polyvinyl alcohol	5–11 Yellow to reddish brown (CR) Pinkish purple to blue to green (ATH)	4%(*v*/*v*) mixture of curcumin and anthocyanin solution at a ratio of 8:2 (*v*/*v*)	[123]
Fish (Hair tail) and shrimp	Curcumin	Curcuma longa	Chitosan (CS)/oxidized chitin nanocrystal (O-ChNCs)	3–10 Yellow to orange red	10% (*w*/*w*, CS and O-ChNCs basis)	[124]
Fish (Rainbow trout fillet)	Alizarin	Roots of Madder family plants	Starch-cellulose	2–11 Yellow to purple	1% (*w*/*v*, mixed starch/glycerol basis)	[26]
Shrimp	Betalains	Hylocereus polyrhizus	Starch/polyvinyl alcohol	3–12 Red (pH ≤ 7) to orange (pH = 8–9) and to yellow (pH = 10–12)	0.25, 0.50 and 1.00% (*w*/*w*, starch basis)	[125]
Shrimp	Anthocyanin	Red rose	Polyvinyl alcohol/okra mucilage polysaccharide	2–12 Red (pH = 2) to pink (pH= 3–5) to blue (pH= 7–10) and to yellow green (pH = 12)	1, 2, 3, and 4% (*w*/*w*, based on PVA)	[126]
Shrimp	Anthocyanin	Echium amoenum flowers	Bacterial cellulose	2–12 Red to yellow	1:1 dilution of extract solution	[127]
Milk	Anthocyanin	Mulberry	κ-carrageenan	2–13 Red to purple and to gray	1.5, 2.5, 3.5, and 4.5% (*w*/*w*, κ-carrageenan basis)	[128]
Milk	Anthocyanin	Grape skins	Tara gum/cellulose	1–10 Bright red to dark green	5 g/100 g, 10 g/ 100 g, and 15 g/100 g (tara gum basis)	[129]
Milk	Anthocyanin	Red cabbage	Polyvinyl alcohol/ starch	No date	No date	[130]
Cheese	Anthocyanin	Red Cabbage	Polyvinylpyrrolidone/CMC/Bacterial cellulose/Guar gum	1–14 Reddish (acidic) to blue (neutral) to green and to yellow (alkaline)	No date	[131]

**Table 3:** The color variations of natural-colorants-based indicators used to monitor food freshness.

Indicator	Food	pH Values	Color Variation	Reference
Based on starch-cellulose and alizarin dye	Fish (rainbow trout fillet) 4 °C	2–11	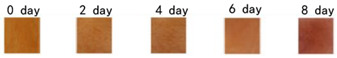	[26]
Based on starch/polyvinyl alcohol and roselle anthocyanins	Fish (4 °C)	2–12	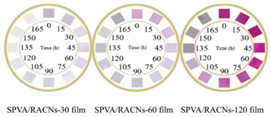	[135]
Based on polyvinyl alcohol/sodium carboxymethyl cellulose and red cabbage anthocyanin	Pork (25 °C)	2–12	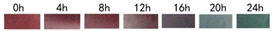	[5]
Based on polyvinyl alcohol/okra mucilage polysaccharide and rose anthocyanins	Shrimp	2–12	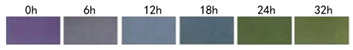	[126]
Based on *κ*-carrageenan and anthocyanins	Milk	2–10	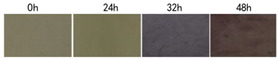	[136]
Based on cellulose nanofibers and blueberry anthocyanin	Lychees	2–12	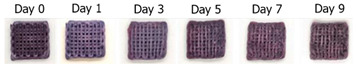	[137]
Based on polyvinyl alcohol/glucomannan and anthocyanins	Banana	3–8	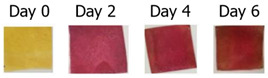	[138]

## Data Availability

Not applicable.

## References

[1] Liu D., Yang L., Shang M., Zhong Y. (2019). Research progress of packaging indicating materials for real-time monitoring of food quality. Mater. Express.

[2] Han J.W., Ruiz-Garcia L., Qian J.P., Yang X.-T. (2018). Food packaging: A comprehensive review and future trends. Compr. Rev. Food. Sci. Food Saf..

[3] Steenis N.D., van Herpen E., van der Lans I.A., Ligthart T.N., van Trijp H.C.M. (2017). Consumer response to packaging design: The role of packaging materials and graphics in sustainability perceptions and product evaluations. J. Clean. Prod..

[4] Balbinot-Alfaro E., Craveiro D.V., Lima K.O., Gouveia Costa H.L., Lopes D.R., Prentice C. (2019). Intelligent packaging with pH indicator potential. Food Eng. Rev..

[5] Liu D., Cui Z., Shang M., Zhong Y. (2021). A colorimetric film based on polyvinyl alcohol/sodium carboxymethyl cellulose incorporated with red cabbage anthocyanin for monitoring pork freshness. Food Packag. Shelf Life.

[6] Fernandez-Pan I., Carrion-Granda X., Mate J.I. (2014). Antimicrobial efficiency of edible coatings on the preservation of chicken breast fillets. Food Control.

[7] Sanches-Silva A., Costa D., Albuquerque T.G., Buonocore G.G., Ramos F., Castilho M.C., Machado A.V., Costa H.S. (2014). Trends in the use of natural antioxidants in active food packaging: A review. Food Addit. Contam. Part A—Chem..

[8] Zhao C.J., Han J.W., Yang X.T., Qian J.P., Fan B.L. (2016). A review of computational fluid dynamics for forced-air cooling process. Appl. Energy.

[9] Poyatos-Racionero E., Vicente Ros-Lis J., Vivancos J.L., Martinez-Manez R. (2018). Recent advances on intelligent packaging as tools to reduce food waste. J. Clean. Prod..

[10] Sohail M., Sun D.W., Zhu Z. (2018). Recent developments in intelligent packaging for enhancing food quality and safety. Crit. Rev. Food Sci. Nutr..

[11] Kuswandi B., Wicaksono Y., Abdullah A., Heng L.Y., Ahmad M. (2011). Smart packaging: Sensors for monitoring of food quality and safety. Sens. Instrum. Food Qual. Saf..

[12] Fang Z., Zhao Y., Warner R.D., Johnson S.K. (2017). Active and intelligent packaging in meat industry. Trends Food Sci. Technol..

[13] Heising J.K., Dekker M., Bartels P.V., Van Boekel M.A.J.S. (2014). Monitoring the quality of perishable foods: Opportunities for intelligent packaging. Crit. Rev. Food Sci. Nutr..

[14] Ghaani M., Cozzolino C.A., Castelli G., Farris S. (2016). An overview of the intelligent packaging technologies in the food sector. Trends Food Sci. Technol..

[15] Choi I., Lee J.Y., Lacroix M., Han J. (2017). Intelligent pH indicator film composed of agar/potato starch and anthocyanin extracts from purple sweet potato. Food Chem..

[16] Ezati P., Tajik H., Moradi M. (2019). Fabrication and characterization of alizarin colorimetric indicator based on cellulose-chitosan to monitor the freshness of minced beef. Sens. Actuator B—Chem..

[17] Realini C.E., Marcos B. (2014). Active and intelligent packaging systems for a modern society. Meat Sci..

[18] Pourjavaher S., Almasi H., Meshkini S., Pirsa S., Parandi E. (2017). Development of a colorimetric pH indicator based on bacterial cellulose nanofibers and red cabbage (*Brassica oleraceae*) extract. Carbohydr. Polym..

[19] Abolghasemi M.M., Sobhi M., Piryaei M. (2016). Preparation of a novel green optical pH sensor based on immobilization of red grape extract on bioorganic agarose membrane. Sens. Actuator B—Chem..

[20] Jafarzadeh S., Nafchi A.M., Salehabadi A., Oladzad-abbasabadi N., Jafari S.M. (2021). Application of bio-nanocomposite films and edible coatings for extending the shelf life of fresh fruits and vegetables. Adv. Colloid Interface Sci..

[21] Kobylewski S., Jacobson M.F. (2012). Toxicology of food dyes. Int. J. Occup. Environ. Health.

[22] Koosha M., Hamedi S. (2019). Intelligent chitosan/PVA nanocomposite films containing black carrot anthocyanin and bentonite nanoclays with improved mechanical, thermal and antibacterial properties. Prog. Org. Coat..

[23] Liu J., Wang H., Wang P., Guo M., Jiang S., Li X., Jiang S. (2018). Films based on kappa-carrageenan incorporated with curcumin for freshness monitoring. Food Hydrocoll..

[24] Bekhit A.E.-D.A., Giteru S.G., Holman B.W.B., Hopkins D.L. (2021). Total volatile basic nitrogen and trimethylamine in muscle foods: Potential formation pathways and effects on human health. Compr. Rev. Food. Sci. Food Saf..

[25] Roy S., Rhim J.W. (2020). Carboxymethyl cellulose-based antioxidant and antimicrobial active packaging film incorporated with curcumin and zinc oxide. Int. J. Biol. Macromol..

[26] Ezati P., Tajik H., Moradi M., Molaei R. (2019). Intelligent pH-sensitive indicator based on starch-cellulose and alizarin dye to track freshness of rainbow trout fillet. Int. J. Biol. Macromol..

[27] Rotariu L., Lagarde F., Jaffrezic-Renault N., Bala C. (2016). Electrochemical biosensors for fast detection of food contaminants trends and perspective. TrAC—Trends Anal. Chem..

[28] Kim J., Moreira R., Castell-Perez E. (2010). Simulation of pathogen inactivation in whole and fresh-cut cantaloupe (*Cucumis melo*) using electron beam treatment. J. Food Eng..

[29] Yong H., Wang X., Zhang X., Liu Y., Qin Y., Liu J. (2019). Effects of anthocyanin-rich purple and black eggplant extracts on the physical, antioxidant and pH-sensitive properties of chitosan film. Food Hydrocoll..

[30] Vandendriessche T., Nicolai B.M., Hertog M.L.A.T.M. (2013). Optimization of hs spme fast gc-ms for high-throughput analysis of strawberry aroma. Food Anal. Meth..

[31] Hui G., Jin J., Deng S., Ye X., Zhao M., Wang M., Ye D. (2015). Winter jujube (zizyphus jujuba mill.) quality forecasting method based on electronic nose. Food Chem..

[32] Zhang Z., Li G., Luo L., Chen G. (2010). Study on seafood volatile profile characteristics during storage and its potential use for freshness evaluation by headspace solid phase microextraction coupled with gas chromatography-mass spectrometry. Anal. Chim. Acta.

[33] Xu K., Lu X., Wang Q., Ma M. (2015). Online automatic grading of salted eggs based on machine vision. Int. J. Agric. Biol. Eng..

[34] Han F., Huang X., Teye E., Gu F., Gu H. (2014). Nondestructive detection of fish freshness during its preservation by combining electronic nose and electronic tongue techniques in conjunction with chemometric analysis. Anal. Methods.

[35] Puligundla P., Jung J., Ko S. (2012). Carbon dioxide sensors for intelligent food packaging applications. Food Control.

[36] Brockgreitens J., Abbas A. (2016). Responsive food packaging: Recent progress and technological prospects. Compr. Rev. Food Sci. F..

[37] Müller P., Schmid M. (2019). Intelligent packaging in the food sector: A brief overview. Foods.

[38] Alizadeh-Sani M., Mohammadian E., Rhim J.W., Jafari S.M. (2020). pH-sensitive (halochromic) smart packaging films based on natural food colorants for the monitoring of food quality and safety. Trends Food Sci. Technol..

[39] Suslick K.S., Rakow N.A., Sen A. (2004). Colorimetric sensor arrays for molecular recognition. Tetrahedron.

[40] Munir S., Hu Y., Liu Y., Xiong S. (2019). Enhanced properties of silver carp surimi-based edible films incorporated with pomegranate peel and grape seed extracts under acidic condition. Food Packag. Shelf.

[41] Chowdhury S., Teoh Y.L., Ong K.M., Zaidi N.S.R., Mah S.K. (2020). Poly (vinyl) alcohol crosslinked composite packaging film containing gold nanoparticles on shelf life extension of banana. Food Packag. Shelf.

[42] De Oliveira Filho J.G., Braga A.R.C., de Oliveira B.R., Gomes F.P., Moreira V.L., Pereira V.A.C., Egea M.B. (2021). The potential of anthocyanins in smart, active, and bioactive eco-friendly polymer-based films: A review. Food Res. Int..

[43] Lopez-Gomez A., Cerdan-Cartagena F., Suardiaz-Muro J., Boluda-Aguilar M., Esther Hernandez-Hernandez M., Angeles Lopez-Serrano M., Lopez-Coronado J. (2015). Radiofrequency identification and surface acoustic wave technologies for developing the food intelligent packaging concept. Food Eng. Rev..

[44] Fleischmann C., Cheng J., Tabatabai M., Ritter H. (2012). Extended applicability of classical phenolphthalein: Color changing polymeric materials derived from pH-sensitive acrylated phenolphthalein derivatives. Macromolecules.

[45] Shukla V., Kandeepan G., Vishnuraj M.R. (2015). Development of on-package indicator sensor for real-time monitoring of buffalo meat quality during refrigeration storage. Food Anal. Meth..

[46] Gavrilenko N.A., Saranchina N.V., Sukhanov A.V., Fedan D.A. (2018). Reversible pH-sensitive element based on bromocresol purple immobilized into the polymethacrylate matrix. Mendeleev Commun..

[47] Zhang Y., Lim L.-T. (2018). Colorimetric array indicator for nh3 and co2 detection. Sens. Actuator B—Chem..

[48] Pacquit A., Lau K.T., McLaughlin H., Frisby J., Quilty B., Diamond D. (2006). Development of a volatile amine sensor for the monitoring of fish spoilage. Talanta.

[49] Shao P., Liu L., Yu J., Zheng L., Sun P. (2022). Novel aldehyde sensitive bio-based colorimetric film for kiwi fruit freshness monitoring. LWT.

[50] Kuswandi B., Jayus, Oktaviana R., Abdullah A., Heng L.Y. (2014). A novel on-package sticker sensor based on methyl red for real-time monitoring of broiler chicken cut freshness. Packag. Technol. Sci..

[51] Kuswandi B., Damayanti F., Jayus, Abdullah A., Heng L.Y. (2015). Simple and low-cost on-package sticker sensor based on litmus paper for real-time monitoring of beef freshness. J. Math. Fundam. Sci..

[52] Nopwinyuwong A., Trevanich S., Suppakul P. (2010). Development of a novel colorimetric indicator label for monitoring freshness of intermediate-moisture dessert spoilage. Talanta.

[53] Rukchon C., Nopwinyuwong A., Trevanich S., Jinkarn T., Suppakul P. (2014). Development of a food spoilage indicator for monitoring freshness of skinless chicken breast. Talanta.

[54] Kuswandi B., Nurfawaidi A. (2017). On-package dual sensors label based on ph indicators for real-time monitoring of beef. Food Control.

[55] Hu Z., Wang H., Li L., Wang Q., Jiang S., Chen M., Li X., Jiang S. (2021). Ph-responsive antibacterial film based polyvinyl alcohol/poly (acrylic acid) incorporated with aminoethyl-phloretin and application to pork preservation. Food Res. Int..

[56] Huang X.w., Zou X.b., Shi J.y., Li Z.h., Zhao J.w. (2018). Colorimetric sensor arrays based on chemo-responsive dyes for food odor visualization. Trends Food Sci. Technol..

[57] Feketea G., Tsabouri S. (2017). Common food colorants and allergic reactions in children: Myth or reality?. Food Chem..

[58] Delgado-Vargas F., Jimenez A.R., Paredes-Lopez O. (2000). Natural pigments: Carotenoids, anthocyanins, and betalains-characteristics, biosynthesis, processing, and stability. Crit. Rev. Food Sci. Nutr..

[59] Ju Z.Y., Howard L.R. (2003). Effects of solvent and temperature on pressurized liquid extraction of anthocyanins and total phenolics from dried red grape skin. J. Agric. Food Chem..

[60] Vatai T., Skerget M., Knez Z. (2009). Extraction of phenolic compounds from elder berry and different grape marc varieties using organic solvents and/or supercritical carbon dioxide. J. Food Eng..

[61] Jafari S.M., Khazaei K.M., Assadpour E. (2019). Production of a natural color through microwave-assisted extraction of saffron tepal’s anthocyanins. Food Sci. Nutr..

[62] Andretta R., Luchese C.L., Tessaro I.C., Spada J.C. (2019). Development and characterization of pH-indicator films based on cassava starch and blueberry residue by thermocompression. Food Hydrocoll..

[63] Roeck F., Barsan N., Weimar U. (2008). Electronic nose: Current status and future trends. Chem. Rev..

[64] Wells N., Yusufu D., Mills A. (2019). Colourimetric plastic film indicator for the detection of the volatile basic nitrogen compounds associated with fish spoilage. Talanta.

[65] Li S., Mu B., Wang X., Kang Y., Wang A. (2019). A comparative study on color stability of anthocyanin hybrid pigments derived from 1d and 2d clay minerals. Materials.

[66] Zhang X., Lu S., Chen X. (2014). A visual pH sensing film using natural dyes from bauhinia blakeana dunn. Sens. Actuator B-Chem..

[67] Wallace T.C., Giusti M.M. (2019). Anthocyanins-nature’s bold, beautiful, and health-promoting colors. Foods.

[68] Castaneda-Ovando A., de Lourdes Pacheco-Hernandez M., Elena Paez-Hernandez M., Rodriguez J.A., Andres Galan-Vidal C. (2009). Chemical studies of anthocyanins: A review. Food Chem..

[69] Smeriglio A., Barreca D., Bellocco E., Trombetta D. (2016). Chemistry, pharmacology and health benefits of anthocyanins. Phytother. Res..

[70] Moradi M., Tajik H., Almasi H., Forough M., Ezati P. (2019). A novel pH-sensing indicator based on bacterial cellulose nanofibers and black carrot anthocyanins for monitoring fish freshness. Carbohydr. Polym..

[71] Romero A., Sharp J.L., Dawson P.L., Darby D., Cooksey K. (2021). Evaluation of two intelligent packaging prototypes with a ph indicator to determine spoilage of cow milk. Food Packag. Shelf Life.

[72] Halasz K., Csoka L. (2018). Black chokeberry (*Aronia melanocarpa*) pomace extract immobilized in chitosan for colorimetric ph indicator film application. Food Packag. Shelf Life.

[73] Liang T., Zhang Z., Jing P. (2019). Black rice anthocyanins embedded in self-assembled chitosan/chondroitin sulfate nanoparticles enhance apoptosis in hct-116 cells. Food Chem..

[74] Jakobek L. (2015). Interactions of polyphenols with carbohydrates, lipids and proteins. Food Chem..

[75] Koh J., Xu Z., Wicker L. (2020). Binding kinetics of blueberry pectin-anthocyanins and stabilization by non-covalent interactions. Food Hydrocoll..

[76] Ezati P., Rhim J.-W. (2020). pH-responsive pectin-based multifunctional films incorporated with curcumin and sulfur nanoparticles. Carbohydr. Polym..

[77] Roy S., Rhim J.-W. (2021). Fabrication of carboxymethyl cellulose/agar-based functional films hybridized with alizarin and grapefruit seed extract. ACS Appl. Bio Mater..

[78] Cvek M., Paul U.C., Zia J., Mancini G., Sedlarik V., Athanassiou A. (2022). Biodegradable films of PLA/PPC and curcumin as packaging materials and smart indicators of food spoilage. ACS Appl. Mater. Interfaces.

[79] Lee W.-H., Loo C.-Y., Bebawy M., Luk F., Mason R.S., Rohanizadeh R. (2013). Curcumin and its derivatives: Their application in neuropharmacology and neuroscience in the 21st century. Curr. Neuropharmacol..

[80] Jovanovic S.V., Steenken S., Boone C.W., Simic M.G. (1999). H-atom transfer is a preferred antioxidant mechanism of curcumin. J. Am. Chem. Soc..

[81] Esatbeyoglu T., Huebbe P., Ernst I.M., Chin D., Wagner A.E., Rimbach G. (2012). Curcumin—from molecule to biological function. Angew. Chem. Int. Ed..

[82] Sun C., Li Y., Han J., Cao B., Yin H., Shi Y. (2019). Enhanced photoelectrical properties of alizarin-based natural dye via structure modulation. Sol. Energy.

[83] Qi X.-N., Che Y.-X., Qu W.-J., Zhang Y.-M., Yao H., Lin Q., Wei T.-B. (2021). Design and fabricating biogenic amine-responsive platform based on self-assembly property of phenazine derivative for visual monitoring of meat spoilage. Sens. Actuators B Chem..

[84] Ezati P., Rhim J.-W. (2020). Ph-responsive chitosan-based film incorporated with alizarin for intelligent packaging applications. Food Hydrocoll..

[85] Polturak G., Aharoni A. (2019). Advances and future directions in betalain metabolic engineering. New Phytol..

[86] Gengatharan A., Dykes G.A., Choo W.S. (2015). Betalains: Natural plant pigments with potential application in functional foods. LWT-Food Sci. Technol..

[87] Khan M.I. (2016). Stabilization of betalains: A review. Food Chem..

[88] Herbach K.M., Stintzing F.C., Carle R. (2006). Stability and color changes of thermally treated betanin, phyllocactin, and hylocerenin solutions. J. Agric. Food Chem..

[89] Gandía-Herrero F., Escribano J., García-Carmona F. (2016). Biological activities of plant pigments betalains. Crit. Rev. Food Sci. Nutr..

[90] Dong H., Ling Z., Zhang X., Zhang X., Ramaswamy S., Xu F. (2020). Smart colorimetric sensing films with high mechanical strength and hydrophobic properties for visual monitoring of shrimp and pork freshness. Sens. Actuators B Chem..

[91] Huang S., Xiong Y., Zou Y., Dong Q., Ding F., Liu X., Li H. (2019). A novel colorimetric indicator based on agar incorporated with arnebia euchroma root extracts for monitoring fish freshness. Food Hydrocoll..

[92] Yoshida C.M., Maciel V.B.V., Mendonça M.E.D., Franco T.T. (2014). Chitosan biobased and intelligent films: Monitoring pH variations. LWT-Food Sci. Technol..

[93] Wu D., Zhang M., Chen H., Bhandari B. (2021). Freshness monitoring technology of fish products in intelligent packaging. Crit. Rev. Food Sci. Nutr..

[94] Kuswandi B., Restyana A., Abdullah A., Heng L.Y., Ahmad M. (2012). A novel colorimetric food package label for fish spoilage based on polyaniline film. Food Control.

[95] Wang W., Li M., Li H., Liu X., Guo T., Zhang G., Xiong Y. (2018). A renewable intelligent colorimetric indicator based on polyaniline for detecting freshness of tilapia. Packag. Technol. Sci..

[96] Zhai X., Wang X., Zhang J., Yang Z., Sun Y., Li Z., Huang X., Holmes M., Gong Y., Povey M. (2020). Extruded low density polyethylene-curcumin film: A hydrophobic ammonia sensor for intelligent food packaging. Food Packag. Shelf Life.

[97] Roy S., Rhim J.-W. (2020). Preparation of carbohydrate-based functional composite films incorporated with curcumin. Food Hydrocoll..

[98] Mannozzi C., Tylewicz U., Chinnici F., Siroli L., Rocculi P., Dalla Rosa M., Romani S. (2018). Effects of chitosan based coatings enriched with procyanidin by-product on quality of fresh blueberries during storage. Food Chem..

[99] Kumar A., Negi Y.S., Choudhary V., Bhardwaj N.K. (2014). Characterization of cellulose nanocrystals produced by acid-hydrolysis from sugarcane bagasse as agro-waste. J. Mater. Phys. Chem..

[100] Priyadarshi R., Kim S.-M., Rhim J.-W. (2021). Pectin/pullulan blend films for food packaging: Effect of blending ratio. Food Chem..

[101] Kerry J., Butler P. (2008). Smart Packaging Technologies for Fast Moving Consumer Goods.

[102] Trouillas P., Sancho-García J.C., De Freitas V., Gierschner J., Otyepka M., Dangles O. (2016). Stabilizing and modulating color by copigmentation: Insights from theory and experiment. Chem. Rev..

[103] Koh J., Xu Z., Wicker L. (2020). Blueberry pectin and increased anthocyanins stability under in vitro digestion. Food Chem..

[104] Pereira P.F., Andrade C.T. (2017). Optimized pH-responsive film based on a eutectic mixture-plasticized chitosan. Carbohydr. Polym..

[105] Mills A., Wild L., Chang Q. (1995). Plastic colorimetric film sensors for gaseous ammonia. Mikrochim. Acta.

[106] Yildiz E., Sumnu G., Kahyaoglu L.N. (2021). Monitoring freshness of chicken breast by using natural halochromic curcumin loaded chitosan/peo nanofibers as an intelligent package. Int. J. Biol. Macromol..

[107] Uranga J., Etxabide A., Guerrero P., de la Caba K. (2018). Development of active fish gelatin films with anthocyanins by compression molding. Food Hydrocoll..

[108] Gaviria Y.A.R., Palencia N.S.N., Capello C., Trevisol T.C., Monteiro A.R., Valencia G.A. (2021). Nanostructured pH-indicator films based on cassava starch, laponite, and jambolan (*syzygium cumini*) fruit manufactured by thermo-compression. Starch-Stärke.

[109] He C., Zhang M., Guo C. (2020). 4d printing of mashed potato/purple sweet potato puree with spontaneous color change. Innov. Food Sci. Emerg. Technol..

[110] Chen H., Wang J., Cheng Y., Wang C., Liu H., Bian H., Pan Y., Sun J., Han W. (2019). Application of protein-based films and coatings for food packaging: A review. Polymers.

[111] Zhao L., Duan G., Zhang G., Yang H., He S., Jiang S. (2020). Electrospun functional materials toward food packaging applications: A review. Nanomaterials.

[112] Topuz F., Uyar T. (2020). Antioxidant, antibacterial and antifungal electrospun nanofibers for food packaging applications. Food Res. Int..

[113] Liu Y.J., Tan J., Yu S.Y., Yousefzadeh M., Lyu T.t., Jiao Z.W., Li H.y., Ramakrishna S. (2020). High-efficiency preparation of polypropylene nanofiber by melt differential centrifugal electrospinning. J. Appl. Polym. Sci..

[114] Wang G., Sun X., Bai J., Han L. (2019). Preparation of Fe–C nanofiber composites by metal organic complex and potential application in supercapacitors. J. Mater. Sci. Mater. Electron..

[115] Wortmann M., Frese N., Sabantina L., Petkau R., Kinzel F., Gölzhäuser A., Moritzer E., Hüsgen B., Ehrmann A. (2019). New polymers for needleless electrospinning from low-toxic solvents. Nanomaterials.

[116] Yoon J.W., Park Y., Kim J., Park C.H. (2017). Multi-jet electrospinning of polystyrene/polyamide 6 blend: Thermal and mechanical properties. Fash. Text..

[117] Liu Y., Wang S., Lan W., Qin W. (2017). Fabrication and testing of PVA/chitosan bilayer films for strawberry packaging. Coatings.

[118] Langroodi A.M., Tajik H., Mehdizadeh T., Moradi M., Kia E.M., Mahmoudian A. (2018). Effects of sumac extract dipping and chitosan coating enriched with zataria multiflora boiss oil on the shelf-life of meat in modified atmosphere packaging. LWT.

[119] Phuhongsung P., Zhang M., Bhandari B. (2020). 4d printing of products based on soy protein isolate via microwave heating for flavor development. Food Res. Int..

[120] Sun G., Chi W., Zhang C., Xu S., Li J., Wang L. (2019). Developing a green film with pH-sensitivity and antioxidant activity based on ĸ-carrageenan and hydroxypropyl methylcellulose incorporating prunus maackii juice. Food Hydrocoll..

[121] Luchese C.L., Abdalla V.F., Spada J.C., Tessaro I.C. (2018). Evaluation of blueberry residue incorporated cassava starch film as pH indicator in different simulants and foodstuffs. Food Hydrocoll..

[122] Goodarzi M.M., Moradi M., Tajik H., Forough M., Ezati P., Kuswandi B. (2020). Development of an easy-to-use colorimetric pH label with starch and carrot anthocyanins for milk shelf life assessment. Int. J. Biol. Macromol..

[123] Chen H.-z., Zhang M., Bhandari B., Yang C.-h. (2020). Novel pH-sensitive films containing curcumin and anthocyanins to monitor fish freshness. Food Hydrocoll..

[124] Wu C., Sun J., Chen M., Ge Y., Ma J., Hu Y., Pang J., Yan Z. (2019). Effect of oxidized chitin nanocrystals and curcumin into chitosan films for seafood freshness monitoring. Food Hydrocoll..

[125] Qin Y., Liu Y., Zhang X., Liu J. (2020). Development of active and intelligent packaging by incorporating betalains from red pitaya (hylocereus polyrhizus) peel into starch/polyvinyl alcohol films. Food Hydrocoll..

[126] Kang S., Wang H., Xia L., Chen M., Li L., Cheng J., Li X., Jiang S. (2020). Colorimetric film based on polyvinyl alcohol/okra mucilage polysaccharide incorporated with rose anthocyanins for shrimp freshness monitoring. Carbohydr. Polym..

[127] Mohammadalinejhad S., Almasi H., Moradi M. (2020). Immobilization of echium amoenum anthocyanins into bacterial cellulose film: A novel colorimetric pH indicator for freshness/spoilage monitoring of shrimp. Food Control.

[128] Liu Y., Qin Y., Bai R., Zhang X., Yuan L., Liu J. (2019). Preparation of pH-sensitive and antioxidant packaging films based on kappa-carrageenan and mulberry polyphenolic extract. Int. J. Biol. Macromol..

[129] Ma Q., Wang L. (2016). Preparation of a visual pH-sensing film based on tara gum incorporating cellulose and extracts from grape skins. Sens. Actuators B Chem..

[130] Mustafa P., Niazi M.B., Jahan Z., Samin G., Hussain A., Ahmed T., Naqvi S.R. (2020). PVA/starch/propolis/anthocyanins rosemary extract composite films as active and intelligent food packaging materials. J. Food Saf..

[131] Bandyopadhyay S., Saha N., Zandraa O., Pummerová M., Sáha P. (2020). Essential oil based pvp-cmc-bc-gg functional hydrogel sachet for ‘cheese’: Its shelf life confirmed with anthocyanin (isolated from red cabbage) bio stickers. Foods.

[132] Becerril R., Nerín C., Silva F. (2021). Bring some colour to your package: Freshness indicators based on anthocyanin extracts. Trends Food Sci. Technol..

[133] Jiang G., Hou X., Zeng X., Zhang C., Wu H., Shen G., Li S.S., Luo Q.Y., Li M.l., Liu X.Y. (2020). Preparation and characterization of indicator films from carboxymethyl-cellulose/starch and purple sweet potato (*Ipomoea batatas* (L.) lam) anthocyanins for monitoring fish freshness. Int. J. Biol. Macromol..

[134] Qin Y., Liu Y., Yong H., Liu J., Zhang X., Liu J. (2019). Preparation and characterization of active and intelligent packaging films based on cassava starch and anthocyanins from *Lycium ruthenicum* Murr. Int. J. Biol. Macromol..

[135] Zhai X., Shi J., Zou X., Wang S., Jiang C., Zhang J., Huang X., Zhang W., Holmes M. (2017). Novel colorimetric films based on starch/polyvinyl alcohol incorporated with roselle anthocyanins for fish freshness monitoring. Food Hydrocoll..

[136] Liu J., Wang H., Guo M., Li L., Chen M., Jiang S., Li X., Jiang S. (2019). Extract from lycium ruthenicum murr. Incorporating κ-carrageenan colorimetric film with a wide pH–sensing range for food freshness monitoring. Food Hydrocoll..

[137] Zhou W., Wu Z., Xie F., Tang S., Fang J., Wang X. (2021). 3d printed nanocellulose-based label for fruit freshness keeping and visual monitoring. Carbohydr. Polym..

[138] Kurnianto M., Poerwanto B., Wahyono A., Apriliyanti M., Lestari I. (2020). Monitoring of Banana Deteriorations using Intelligent-packaging Containing Brazilien Extract (*Caesalpina sappan* L.). IOP Conference Series: Earth and Environmental Science.

[139] Stoll L., Costa T.M.H., Jablonski A., Flôres S.H., de Oliveira Rios A. (2016). Microencapsulation of anthocyanins with different wall materials and its application in active biodegradable films. Food Bioprocess Technol..

[140] Kurek M., Hlupić L., Ščetar M., Bosiljkov T., Galić K. (2019). Comparison of two pH responsive color changing bio-based films containing wasted fruit pomace as a source of colorants. J. Food Sci..

[141] Gómez I., Janardhanan R., Ibañez F.C., Beriain M.J. (2020). The effects of processing and preservation technologies on meat quality: Sensory and nutritional aspects. Foods.

[142] Othman M., Yusup A.A., Zakaria N., Khalid K. (2018). In Bio-polymer chitosan and corn starch with extract of hibiscus rosa-sinensis (hibiscus) as ph indicator for visually-smart Food Packaging. AIP Conf. Proc..

[143] Dudnyk I., Janeček E.-R., Vaucher-Joset J., Stellacci F. (2018). Edible sensors for meat and seafood freshness. Sens. Actuators B Chem..

[144] Zhang J., Zou X., Zhai X., Huang X., Jiang C., Holmes M. (2019). Preparation of an intelligent pH film based on biodegradable polymers and roselle anthocyanins for monitoring pork freshness. Food Chem..

[145] Zhang J., Huang X., Zou X., Shi J., Zhai X., Liu L., Li Z., Holmes M., Gong Y., Povey M. (2021). A visual indicator based on curcumin with high stability for monitoring the freshness of freshwater shrimp, *Macrobrachium rosenbergii*. J. Food Eng..

[146] Zhang X., Zhao Y., Shi Q., Zhang Y., Liu J., Wu X., Fang Z. (2021). Development and characterization of active and pH-sensitive films based on psyllium seed gum incorporated with free and microencapsulated mulberry pomace extracts. Food Chem..

[147] Karaman A., Özer B., Pascall M.A., Alvarez V. (2015). Recent advances in dairy packaging. Food Rev. Int..

[148] Pereira V.A., de Arruda I.N.Q., Stefani R. (2015). Active chitosan/PVA films with anthocyanins from brassica oleraceae (red cabbage) as time–temperature indicators for application in intelligent food packaging. Food Hydrocoll..

[149] Liu B., Xu H., Zhao H., Liu W., Zhao L., Li Y. (2017). Preparation and characterization of intelligent starch/PVA films for simultaneous colorimetric indication and antimicrobial activity for food packaging applications. Carbohydr. Polym..

[150] Zhai X., Li Z., Zhang J., Shi J., Zou X., Huang X., Zhang D., Sun Y., Yang Z., Holmes M. (2018). Natural biomaterial-based edible and pH-sensitive films combined with electrochemical writing for intelligent food packaging. J. Agric. Food Chem..

[151] Da Rosa C.G., Sganzerla W.G., Maciel M.V.d.O.B., de Melo A.P.Z., da Rosa Almeida A., Nunes M.R., Bertoldi F.C., Barreto P.L.M. (2020). Development of poly (ethylene oxide) bioactive nanocomposite films functionalized with zein nanoparticles. Colloids Surf. A Physicochem. Eng. Asp..

[152] Pavinatto A., de Almeida Mattos A.V., Malpass A.C.G., Okura M.H., Balogh D.T., Sanfelice R.C. (2020). Coating with chitosan-based edible films for mechanical/biological protection of strawberries. Int. J. Biol. Macromol..

[153] Chen H.-z., Zhang M., Bhandari B., Guo Z. (2018). Applicability of a colorimetric indicator label for monitoring freshness of fresh-cut green bell pepper. Postharvest Biol. Technol..

[154] Sganzerla W.G., Ribeiro C.P.P., Uliana N.R., Rodrigues M.B.C., da Rosa C.G., Ferrareze J.P., de Lima Veeck A.P., Nunes M.R. (2021). Bioactive and pH-sensitive films based on carboxymethyl cellulose and blackberry (*Morus nigra* L.) anthocyanin-rich extract: A perspective coating material to improve the shelf life of cherry tomato (*Solanum lycopersicum* L. *Var. Cerasiforme*). Biocatal. Agric. Biotechnol..

[155] Torres-León C., Vicente A.A., Flores-López M.L., Rojas R., Serna-Cock L., Alvarez-Pérez O.B., Aguilar C.N. (2018). Edible films and coatings based on mango (var. Ataulfo) by-products to improve gas transfer rate of peach. LWT.

